# Evaluation of the SII and HALP scores in predicting perinatal outcomes in pregnancies with preterm premature rupture of membranes

**DOI:** 10.1186/s12884-026-09127-9

**Published:** 2026-04-20

**Authors:** Cagdas Nurettin Emeklioglu, Mirac Ozalp, Elif Akkoc Demirel, Simten Genc, Veli Mihmanli

**Affiliations:** 1 Division of Perinatology, Department of Obstetrics and Gynecology, Marmara University Pendik Research and Training Hospital, Fevzi Cakmak, Muhsin Yazicioglu Cd No:10, Pendik, 34899 Istanbul, Turkey; 2 Division of Perinatology, Department of Obstetrics and Gynecology, Liv Ulus Hospital, Istanbul, Turkey; 3 Department of Obstetrics and Gynecology, Prof.Dr. Cemil Tascioglu City Hospital, Istanbul, Turkey; 4 Private Clinic, Istanbul, Turkey

**Keywords:** Systemic immune-inflammation index, SII, HALP score, Neonatal outcomes, Prediction, PPROM

## Abstract

**Background:**

Preterm premature rupture of membranes (PPROM) is a leading cause of preterm birth and neonatal morbidity, with inflammation playing a key role in its pathogenesis. This study aimed to evaluate the predictive value of the Systemic Immune-Inflammation Index (SII) and the Hemoglobin-Albumin-Lymphocyte-Platelet (HALP) score for perinatal outcomes and delivery timing in pregnancies complicated by PPROM.

**Methods:**

This retrospective cross-sectional study included 317 pregnant women who were diagnosed with PPROM between 24 and 36 + 6 weeks gestation, from January 2018 to January 2024. SII and HALP scores were calculated from blood parameters at admission using the following formulas: SII = [platelets(/L) × neutrophils(/L)] / lymphocytes(/L) and HALP = [hemoglobin(g/L) × albumin(g/L) × lymphocytes(/L)] / platelets(/L). Neonatal outcomes (birth weight, APGAR scores, NICU admission) and delivery timing were analyzed. ROC curves and logistic regression models were used to assess predictive performance.

**Results:**

Higher SII values were significantly associated with lower birth weights, lower 1- and 5-minute APGAR scores, and increased NICU admission. The SII showed modest predictive power for NICU admission (AUC: 0.653, cut-off = 1145.75) and strong discriminative ability for predicting delivery within 7 days (AUC: 0.860, cut-off: 1421, sensitivity and specificity: 78%). In contrast, the HALP showed limited and inconsistent discriminative performance and was not significantly associated with adverse outcomes. Multivariate logistic regression confirmed that the SII was as an independent predictor of NICU admission (*p* = 0.012).

**Conclusion:**

The SII is a promising biomarker for predicting adverse neonatal outcomes and the timing of delivery in PPROM patients. A SII value > 1421 may indicate delivery within 7 days, aiding in clinical management. Each 100-unit increase in SII was associated with an increased risk of NICU admission. The HALP score was not a reliable predictor.

**Supplementary Information:**

The online version contains supplementary material available at 10.1186/s12884-026-09127-9.

## Background

Preterm premature rupture of membranes (PPROM) is generally defined as rupture of the amniotic membrane before the 37th week of gestation [1]. PPROM is a well-established risk factor for preterm delivery, accounting for approximately one-third of all preterm births and complicating 1–4.5% of pregnancies worldwide [2–4]. Various factors, alone or in combination, may contribute to membrane rupture during the preterm period. Microbiological and inflammatory processes have been shown to underlie this process; in particular, subclinical infection and localized inflammation can weaken the amnion-chorion junction, leading to premature rupture [5, 6].

Clinical, molecular biological and histological data suggest that inflammation and focal infection play a primary or secondary role in the pathogenesis of PPROM [7–9]. Systemic inflammation is characterized by changes such as neutrophilia, lymphopenia and elevated C-reactive protein (CRP) [10, 11]. However, the sensitivity of isolated changes in these parameters is quite poor. Therefore, owing to its simplicity and accessibility, assessing systemic inflammation via basic hematologic indices has gained attention in recent years. In particular, the Systemic Immune-Inflammation Index (SII), which is derived from complete blood count parameters, and the Hemoglobin-Albumin-Lymphocyte-Platelet (HALP) score, which incorporates serum albumin, have emerged as useful markers of systemic inflammation and prognostic indicators in malignancies, cardiovascular diseases, and inflammation [12–14].

The diagnosis of PPROM is mainly clinical, and identifying biomarkers that can predict neonatal outcomes may be critical for optimizing neonatal care. In the literature, the prognostic role of the HALP score in PPROM has not been investigated yet, and few studies on the SII have focused on different end-points [10, 15].

To overcome this deficiency, this study evaluated the associations of HALP scores and SII values at the time of hospital admission with neonatal outcomes (APGAR scores, birth weights, NICU admissions) in pregnant women diagnosed with PPROM in a retrospective cohort. Furthermore, the performance of these scores in predicting the time of delivery in pregnant women diagnosed with PPROM was evaluated in the same cohort.

## Materials and methods

This retrospective cross-sectional study included 317 pregnant women who presented with complaints of amniotic fluid leakage to the Obstetric Emergency Department at Prof. Dr. Cemil Taşcıoğlu City Hospital between January 2018 and January 2024 and were diagnosed with PPROM. The inclusion criteria were singleton pregnancies between 24 + 0 and 36 + 6 weeks of gestation, maternal age between 18 and 45 years, and hospital admission to the specified department. The study protocol was approved by the local ethics committee (Approval date-number: 06.05.2024, 102). The study was conducted in accordance with the principles of the Declaration of Helsinki.

The exclusion criteria included women with chronic or systemic conditions (e.g., hypertension, diabetes, hypothyroidism), those who presented with fluid leakage but without confirmed membrane rupture, and those presenting before 24 weeks or at 37 weeks or beyond. Furthermore, patients who had received antenatal corticosteroids at another institution prior to their admission were excluded to eliminate potential interference with the inflammatory markers. Additional exclusions were made for patients who delivered elsewhere, left the hospital after initial admission, or had incomplete laboratory data needed to calculate HALP or the SII.

Maternal demographics, gravidity, parity, number of abortions, gestational age at admission, latency from rupture to delivery, and delivery mode were recorded. The neonatal outcomes included birth weight, 1- and 5-minute APGAR scores, and neonatal intensive care unit (NICU) admission. The gestational age of all pregnant women included in the study was confirmed by first trimester ultrasound measurements. To calculate the investigated HALP scores and SII values, the hemoglobin, lymphocyte, neutrophil, leukocyte, platelet, albumin and CRP values in the blood parameters taken at the time of the first admission to the hospital with the complaint of amniotic fluid leakage of these pregnant women were retrieved and recorded from the hospital electronic database. The formula [hemoglobin(g/L) × albumin(g/L) × lymphocytes(/L)] / platelets(/L) was used to calculate the HALP score, whereas the formula [platelets(/L) × neutrophils(/L)] / lymphocytes(/L) was used to calculate the SII. These calculations were conducted in Microsoft Excel for Mac 2019.

All patients underwent sterile speculum examination per the institutional protocol. The diagnosis of PPROM was established upon direct visualization of amniotic fluid leakage from the cervical os during sterile speculum examination. In cases of diagnostic uncertainty, placental alpha microglobulin-1 testing was performed to confirm membrane rupture. All patients were admitted immediately upon rupture recognition. All patients diagnosed with PPROM were hospitalized and managed according to an antenatal care protocol consistent with current guidelines [16, 17]. Following admission, maternal and fetal well-being were monitored, and a standardized antibiotic regimen—comprising single-dose oral azithromycin (1 g), followed by intravenous ampicillin (2 g every 6 h for 48 h), and subsequently oral amoxicillin for 5 days if labor had not ensued—was administered. However, immediate delivery was only planned for pregnant women with overt clinical signs of chorioamnionitis or in cases where fetal well-being was compromised. In the absence of these specific clinical indications, labor was allowed to ensue spontaneously. This approach ensured that the timing of delivery was primarily driven by the biological progression of the pregnancy rather than elective iatrogenic intervention.

### Statistical analysis

Statistical analyses were performed via SPSS 26.0 (IBM Corp., Armonk, NY). There were no missing data for the variables analyzed in the study. The distribution of variables was assessed for normality both visually (histograms, probability plots) and analytically (Kolmogorov-Smirnov/Shapiro-Wilk tests). Normally distributed continuous variables were compared between groups using the Student’s t-test, while non-normally distributed variables were analyzed using the Mann-Whitney U test. Correlations between non-normally distributed data were assessed using Spearman’s rank correlation coefficient. Categorical variables were presented using cross-tabulations and compared using the Chi-square test or Fisher’s exact test, as appropriate.

Receiver operating characteristic (ROC) curves were used to assess the predictive performance of HALP and the SII for NICU admission and delivery within 7 days after amniotic fluid leakage. The 7-day latency threshold was selected based on its established clinical significance in PPROM management, as it represents the optimal window for the therapeutic benefit of antenatal corticosteroids and is a standardized endpoint in obstetric literature for assessing the success of expectant management. In the presence of significant cut-off values, sensitivity, specificity, positive and negative predictive values of these cut-off values were calculated. In the evaluation of the area under the curve (AUC), cases with a type-1 error level less than 0.05 were considered statistically significant. In the multivariate analysis, the independent predictors of the probable outcome were examined via logistic regression analysis using the possible factors identified in the previous analyses. The multivariable logistic regression model was specifically constructed to adjust for primary clinical confounders, most notably gestational age at admission, to verify whether inflammatory indices (SII and HALP) carry independent prognostic weight regardless of the degree of prematurity. The Hosmer-Lemeshow test was used for model fit. For clinical interpretability, SII was additionally analyzed as a rescaled variable (per 100-unit increase) in logistic regression models. Type-1 error levels below 5% were interpreted as statistically significant.

## Results

The study included 317 women diagnosed with PPROM and their infants admitted to the hospital within the specified period. Maternal demographics and clinical characteristics stratified by delivery route are presented in Table 1. Vaginal delivery occurred in 43.8% (*n* = 139) of pregnant women, while the remaining pregnant women underwent cesarean section with various indications. The SII and HALP scores were calculated on the basis of the laboratory parameters at the time of admission, with the complaint of fluid leakage.

**Table 1:** Comparison of maternal characteristics, inflammatory indices, and neonatal outcomes according to the mode of delivery in pregnancies complicated by PPROM

		Delivery method			
		C/S	Vaginal	*p*	
Age	*Mean ± SD*	30,04 ± 6,23	27,07 ± 6,99	0,001*	
Week	*Mean ± SD*	33,05 ± 3,04	33,93 ± 2,29	0,005*	
G	*Q2 (Q1-Q3)*	3 (1–4)	2 (1–4)	0,182**	
P	*Q2 (Q1-Q3)*	1 (0–2)	1 (0–2)	0,036**	
A	*Q2 (Q1-Q3)*	0 (0–1)	0 (0–1)	0,087**	
Hb (g/L)	*Mean ± SD*	113,21 ± 16,93	114,61 ± 15,11	0,443*	
Alb (g/L)	*Mean ± SD*	32,99 ± 3,56	33,69 ± 3,18	0,067*	
LYM (/L)	*Q2 (Q1-Q3)*	1,77 (1,455-2,2325)	2,05 (1,62 − 2,54)	0,002**	
PLT (/L)	*Q2 (Q1-Q3)*	231,5 (190,75–279,75)	230 (187–278)	0,695**	
NTR (/L)	*Q2 (Q1-Q3)*	9,775 (7,335 − 13,31)	9,64 (7,26 − 12,2)	0,446**	
WBC (/µL)	*Mean ± SD*	12,76 ± 4,17	12,74 ± 3,52	0,952*	
CRP (mg/L)	*Q2 (Q1-Q3)*	7,88 (3,84 − 16)	6,55 (3,64 − 11,01)	0,035**	
Fetal Weight (g)	*Mean ± SD*	2376 ± 643	2678 ± 644	0,001*	
APGAR-1	*Mean ± SD*	6,76 ± 1,19	7,12 ± 1,14	0,008*	
APGAR-5	*Mean ± SD*	8,26 ± 0,88	8,63 ± 0,87	0,001*	
HALP score	*Q2 (Q1-Q3)*	29,2 (20,35–37,7775)	36,44 (24,57 − 48,69)	0,001**	
SII	*Q2 (Q1-Q3)*	1217,45 (865,8-1821,4)	1093,87 (730,71-1541,15)	0,005**	
NICU n (%)	*No*	74 (41,6%)	76 (54,7%)	0,021***	
	*Yes*	104 (58,4%)	63 (45,3%)		
Days from PPROM to delivery	*Q2 (Q1-Q3)*	2 (0–9)	1 (0–6)	0,019**	
> 7 days	*n (%)*	51 (28,7%)	24 (17,3%)	0,018***	
≤ 7 days	*n (%)*	127 (71,3%)	115 (82,7%)		

*G* Gravida, *P* Parity, *A* Abortus, *Hb* Hemoglobin, *Alb* Albumin, *LYM* Lymphocytes, *PLT* Platelets, *NTR* Neutrophils, *WBC* Leukocytes, *CRP* C-reactive Protein, *HALP score* Hemoglobin, albumin, lymphocyte, and platelets’ score, *SII* Systemic immune-inflammation index, *NICU* Neonatal Intensive Care Unit. *: Independent sample t-test; **: Mann- Whitney U; ***: Pearson chi-square.

Table 2 presents the correlation analyses between HALP scores, SII values, and various demographic and clinical parameters, along with corresponding Pearson correlation coefficients (r) and significance levels (p). A weak but significant negative correlation was observed between HALP scores and the latency period (*r* = -0.279, *p* = 0.001) and the 1-minute APGAR score (*r* = -0.127, *p* = 0.023). While a moderate positive correlation was identified between SII values and the latency period (*r* = 0.602, *p* = 0.001), significant negative correlations were observed between SII and newborn birth weight (*r* = -0.272, *p* = 0.001), as well as 1-minute (*r* = -0.163, *p* = 0.004) and 5-minute (*r* = − 0.244, *p* = 0.001) APGAR scores.

**Table 2:** Correlation analysis of SII and HALP scores with demographic data and neonatal outcomes

		HALP score	SII
Age	*r*	-0,045	0,071
	p	0,428	0,207
Week	r	-0,067	-0,087
	p	0,236	0,121
G	r	-0,055	-0,004
	p	0,333	0,937
P	r	-0,074	0,018
	p	0,187	0,745
A	r	-0,078	0,093
	p	0,164	0,097
Fetal Weight (g)	r	-0,079	-,272
	p	0,162	0,001**
APGAR-1	r	-,127	-,163
	p	0,023*	0,004**
APGAR-5	r	-0,058	-,244
	p	0,304	0,001**
Latency Period	r	-0,279	0,602
	p	0,001**	0,001**

*G* Gravida, *P* Parity, *A* Abortus

* Correlation is significant at the 0.05 level (2-tailed)

** Correlation is significant at the 0.01 level (2-tailed)

**Fig. 1:**
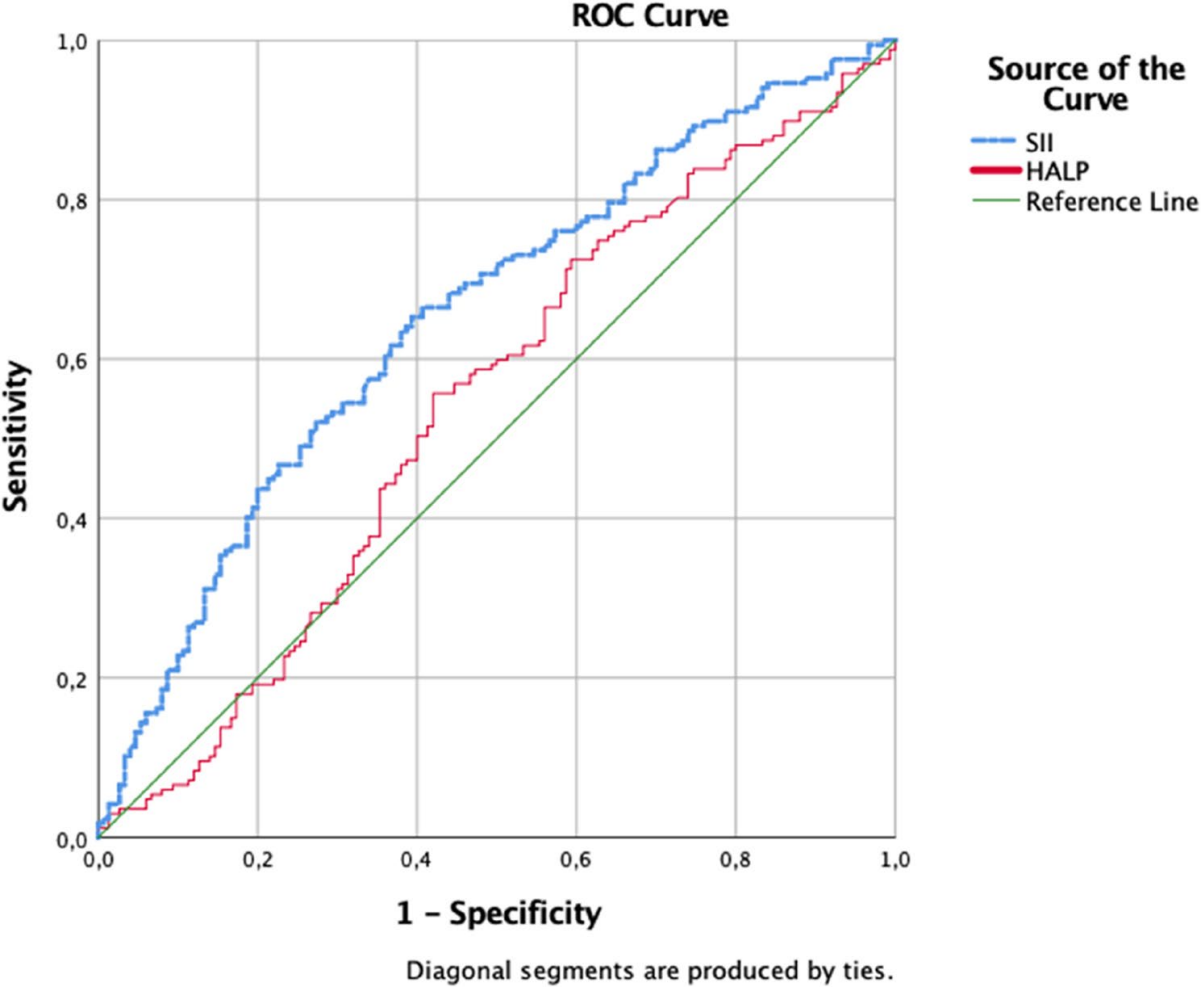
ROC curve plotted for SII and HALP scores in predicting NICU admission. For SII: AUC: 0.653 (95% CI:0.593–0.713, *p* = 0.001), cut-off: 1145.75, sensitivity: 62%, specificity: 63%. For HALP: AUC: 0.542 (95% CI:0.478–0.606, *p* = 0.201), cut-off: 31.12, sensitivity: 56%, specificity: 56%. (AUC, area under the curve; CI, confidence interval; SII, systemic immune-inflammation index; HALP, hemoglobin-albumin-lymphocyte-platelet score)

To assess the discriminatory ability of the SII and HALP scores in predicting NICU admission, ROC curves were generated and the areas under the curve (AUC) are presented in Fig. 1. The AUC for the SII value was 0.653, indicating a statistically significant discriminatory capacity (*p* = 0.001). The optimal SII cut-off value was determined to be 1145.75, yielding a sensitivity of 62% and a specificity of 63%. In contrast, the AUC for the HALP score was calculated as 0.542, indicating no statistically significant discriminative power (*p* = 0.201). At the optimal cut-off value of 31.12, both the sensitivity and specificity were 56% (Fig. 1).

Univariate and multivariate logistic regression analyses of factors associated with NICU admission are presented in Table 3. As expected, gestational age, birth weight, and 1st and 5th minute APGAR scores were significantly associated with NICU admission (all *p* < 0.001). The leukocyte count and CRP level were not significantly associated. While the HALP score was not a significant predictor according to the univariate analysis (OR = 1.004, 95% CI:0.991–1.017; *p* = 0.528), higher SII values were significantly associated with increased NICU admission risk (OR = 1.001, 95% CI:1–1.001; *p* = 0.001). This association remained statistically significant in the multivariate model (OR = 1, 95% CI:1–1.001; *p* = 0.012).

**Table 3:** Logistic regression analysis of variables affecting NICU admission

	Univariate Analysis				Multivariate Analysis			
	B	OR	95% CI	Sig.	B	OR	95% CI	Sig.
Age	0,002	1,002	0,969-1,035	0,924				
Week	-0,325	0,722	0,65 − 0,802	**0**,**001**	-0,31	0,733	0,644-0,835	**0**,**001**
G	0,049	1,05	0,935-1,18	0,408				
P	0,002	1,002	0,848-1,184	0,983				
A	0,125	1,133	0,911-1,41	0,261				
Fetal Weight (g)	-0,003	0,997	0,997-0,998	**0**,**001**				
APGAR-1	-1,317	0,268	0,189-0,379	**0**,**001**				
APGAR-5	-1,526	0,217	0,147-0,322	**0**,**001**	-1,288	0,276	0,181-0,419	**0**,**001**
WBC (/µL)	0,017	1,017	0,961-1,077	0,558				
CRP (mg/L)	0,012	1,012	0,999-1,025	0,078				
HALP	0,004	1,004	0,991-1,017	0,528				
SII	0,001	1,001	1–1,001	**0**,**001**	0,001	1	1–1,001	**0**,**012**

*G* Gravida, *P* Parity, *A* Abortus, *WBC* Leukocytes, *CRP* C-reactive Protein, *HALP score* Hemoglobin, albumin, lymphocyte, and platelets’ score, *SII* Systemic immune-inflammation index. Bold values indicate statistical significance (*p*<0.05)

**Fig. 2:**
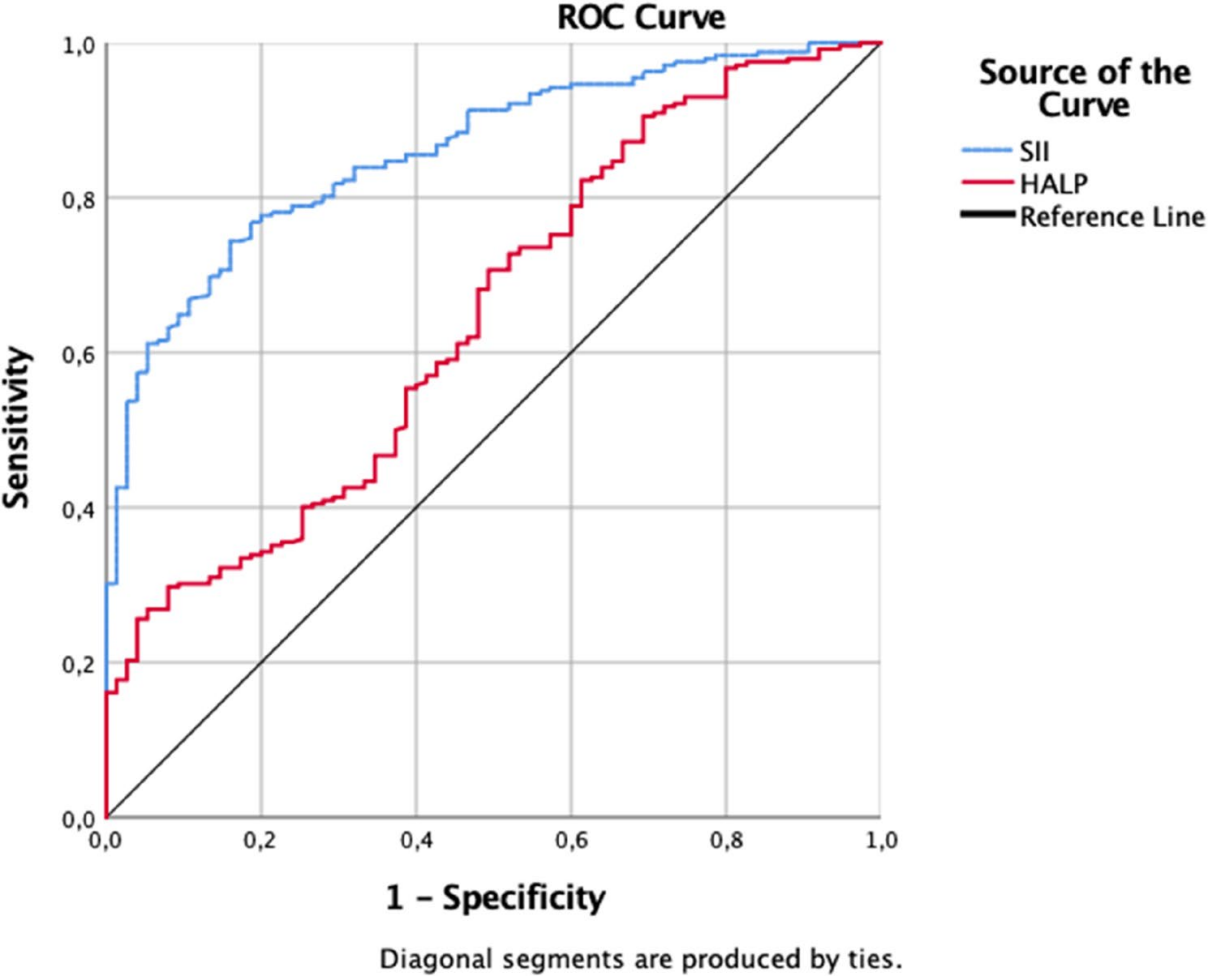
ROC curve plotted for SII and HALP scores for predicting labor within 7 days after PPROM. For SII: AUC: 0.86 (*p* = 0.001), cut-off: 1421, sensitivity: 78%, specificity: 78%. Since the initial ROC analysis for HALP yielded an AUC below 0.5, indicating an inverse association, HALP values were reverse-coded and the analysis was repeated. After reverse coding, for HALP: AUC: 0.647 (*p* = 0.001), cut-off: 29.755, sensitivity: 57.4%, specificity: 57.3%. (AUC, area under the curve; SII, systemic immune-inflammation index; HALP, hemoglobin-albumin-lymphocyte-platelet score)

To assess the ability of the SII and HALP score to predict the occurrence of labor within 7 days following membrane rupture, ROC curve analyses were performed, and the corresponding AUC values are presented in Fig. 2. The SII demonstrated excellent discriminative performance, with an AUC of 0.860. At the optimal cut-off value of 1421, both the sensitivity and specificity were calculated as 78%, indicating strong predictive accuracy. Conversely, the initial ROC analysis for HALP scores yielded an AUC below 0.500, indicating an inverse association with the outcome. Consequently, HALP values were reverse-coded and the analysis was repeated; following this adjustment, the AUC was determined to be 0.647 (Fig. 2).

## Discussion

As PPROM significantly impacts neonatal health, our study evaluated the SII and HALP scores as prognostic markers. Elevated SII values at initial presentation negatively correlated with birth weight and both 1- and 5-minute APGAR scores, potentially offering early insights into the newborn’s condition. Conversely, although HALP scores showed a negative correlation with the 1-minute APGAR score, their lack of association with 5-minute scores and birth interval suggests limited clinical utility for predicting overall neonatal outcomes.

Admission-based ROC analyses indicated that an SII cut-off of 1145.75 significantly predicted NICU risk. While its discriminative ability was modest (AUC:0.653; sensitivity: 62%, specificity: 63%), the SII offers potential as an adjunctive biomarker. Conversely, the HALP score showed poor predictive performance, limiting its clinical utility for discriminating NICU admission risk in this population. The HALP score’s poor performance compared to the SII highlights its potential unsuitability for obstetric populations. Pregnancy-induced plasma expansion causes hemodilution, leading to physiological anemia and hypoalbuminemia. Since hemoglobin and albumin are in the HALP formula’s numerator, these predictable physiological drops may mask acute inflammatory responses in PPROM. Furthermore, NICU admission was utilized as a pragmatic surrogate for clinical burden and resource allocation, reflecting the integrated decision-making process in tertiary care and providing a robust baseline for risk stratification at presentation.

While logistic regression reaffirmed standard predictors like gestational age and birth weight, the SII’s independent and persistent association with NICU risk across both models represents a novel finding. Although the reported odds ratio for a single-unit increase in the SII was close to unity (OR = 1.001, 95% CI:1–1.001; *p* = 0.012), this should be interpreted within the context of the index’s broad numerical range. When evaluated in clinically meaningful increments, a 100-unit increase in the SII reflects approximately a 10% increase in the risk of NICU admission. Therefore, the clinical significance of the SII as a prognostic biomarker is better demonstrated through these substantial increments rather than marginal single-unit changes. This highlights the SII as a robust prognostic biomarker, offering significant potential for optimizing the clinical management of PPROM.

In estimating labor timing following PPROM, the SII demonstrated excellent performance, predicting delivery within 7 days with an AUC of 0.860. An optimal cut-off of 1421 provided 78% sensitivity and specificity, underscoring its strong clinical applicability. Although the SII demonstrated a positive correlation with latency in continuous analysis, its excellent performance in predicting delivery within 7 days (AUC: 0.860) suggests a complex, non-linear relationship between systemic inflammation and the timing of delivery. This apparent discrepancy may reflect the heterogeneous clinical trajectories in PPROM; while an elevated inflammatory burden typically drives rapid progression to labor, it may also be associated with prolonged latency in a subset of patients who do not immediately trigger the labor cascade despite high systemic indices. This suggests that the SII may be more effective as a threshold-based predictor rather than a continuous linear marker for delivery timing. Conversely, the HALP score showed poor discriminative power (AUC: 0.647), indicating limited efficacy for predicting the time of labor in this population.

Many studies in the literature have evaluated the effectiveness of maternal blood parameters and related indices and values in predicting pregnancy outcomes [18–20]. Complications associated with inflammation such as preeclampsia, intrahepatic cholestasis during pregnancy and preterm delivery seem to be the leading subjects of these studies. On the other hand, the SII is a well-recognized inflammatory marker with validated prognostic utility in oncology and cardiovascular medicine [12, 13, 21–24].

In a recent study in the literature, the ability of the SII value and inflammatory markers to predict the time of delivery in pregnant women with PPROM was investigated and it was shown that this prediction could not be achieved with the SII [10]. Tanacan et al. reported a relationship between the SII and poor neonatal outcomes and reported that the SII was a parameter that could be used as an additional parameter to predict neonatal outcomes [15]. While definitions such as respiratory distress syndrome and necrotizing enterocolitis emerged as poor neonatal outcomes in this study, we used variables such as the need for NICU admission, low birth weight and low APGAR score and designed our study with a larger study group than that used in this study. Since the retrospective nature of our study precluded consistent access to data on specific morbidities, NICU admission served as a robust and clinically relevant surrogate for neonatal health. A recent study by Hrubaru et al. revealed that the risk of preterm delivery could be predicted with third trimester HALP scores, but no data concerning neonatal outcomes were reported [25].

To the best of our knowledge, no prior study has specifically investigated the associations between HALP scores and neonatal outcomes in pregnancies complicated by PPROM. This positions our study as the first to explore and report on this relationship in the existing literature. Notably, our findings revealed that elevated SII values at initial presentation are significantly associated with lower birth weights, lower APGAR scores, and higher rates of NICU admission, suggesting the potential utility of the SII as an early prognostic indicator of adverse neonatal outcomes. These results may be particularly valuable in resource-limited settings, where access to advanced neonatal care is constrained and early risk stratification is critical. Nevertheless, larger prospective studies are warranted to validate these findings and to support the integration of the SII and HALP scores into routine clinical decision-making.

This study represents a novel contribution to the literature, as it is the first to evaluate the prognostic role of the HALP score in predicting NICU admission among pregnant women diagnosed with PPROM. Furthermore, this study is the first to concurrently assess both the SII and HALP scores in relation to the timing of delivery in this population. The strengths of this study include an adequate sample size to allow meaningful interpretation, the use of standardized and robust statistical methods, and the simultaneous evaluation of two relatively understudied inflammatory indices. Additionally, the identification of clinically relevant cut-off values enhances the potential for external validation in future multicenter studies. However, several limitations warrant consideration. The retrospective, single-center design restricts generalizability, and the lack of serial measurements precludes assessing dynamic changes in these indices. Additionally, the use of NICU admission as a primary outcome has inherent limitations, as it is a context-dependent marker influenced by institutional policies and gestational age thresholds rather than representing individual neonatal morbidities in isolation. Furthermore, while our study utilized a dichotomous 7-day threshold for delivery timing, we recognize that a time-to-event approach, such as Cox proportional hazards regression, could provide further insights into the relationship between inflammatory indices and the latency period. The proposed SII cut-off values were derived and evaluated within the same cohort. Therefore, these thresholds should be considered exploratory. External validation in independent and larger populations is required before clinical implementation. Future prospective studies incorporating these specific neonatal morbidities alongside serial measurements—particularly near term—may further elucidate the prognostic utility of these indices.

## Conclusion

The SII may serve as a complementary biomarker to existing predictive tools for estimating the risk of NICU admission and other adverse neonatal outcomes in pregnancies complicated by PPROM. While individual unit changes in the SII may appear minimal, its clinical importance is evidenced by substantial risk increments, such as a 10% increase in NICU risk for every 100-unit rise in the index. Notably, an SII value exceeding 1421 at the time of hospital admission appears to be a strong predictor of delivery within the subsequent seven days, highlighting its potential utility in early clinical decision-making.

## Supplementary Information

Supplementary Material 1.

Supplementary Material 2.

## Data Availability

Datasets analyzed or generated during the study is available upon request from corresponding author.

## Abbreviations

PPROM: Preterm premature rupture of membranes
HALP: Hemoglobin-Albumin-Lymphocyte-Platelet
SII: Systemic Immune-Inflammation Index
NICU: Neonatal Intensive Care Unit
CRP: C-reactive Protein
ROC: Receiver Operating Characteristic
AUC: Area Under The Curve
