# Supplementary material for: Evaluation of the SII and HALP scores in predicting perinatal outcomes in pregnancies with preterm premature rupture of membranes
Source: BMC Pregnancy Childbirth. 2026 Apr 20;26:595. doi: 10.1186/s12884-026-09127-9 (PMC13227662; doi:10.1186/s12884-026-09127-9)
Supplement: Supplementary file 1 — Supplementary Material 1. [file 12884_2026_9127_MOESM1_ESM.docx]

**Evaluation of the SII and HALP Scores in Predicting Perinatal Outcomes in Pregnancies with Preterm Premature Rupture of Membranes (PPROM)**

### Cagdas Nurettin Emeklioglu ¹*, Mirac Ozalp ², Elif Akkoc Demirel ³, Simten Genc ⁴, Veli Mihmanli ³

¹ Division of Perinatology, Department of Obstetrics and Gynecology, Marmara University Pendik Research and Training Hospital, Istanbul, Turkey

² Division of Perinatology, Department of Obstetrics and Gynecology, Liv Ulus Hospital, Istanbul, Turkey

³ Department of Obstetrics and Gynecology, Prof.Dr. Cemil Tascioglu City Hospital, Istanbul, Turkey

⁴ Private Clinic, Istanbul, Turkey

*****Corresponding Author: Cagdas Nurettin Emeklioglu

Address: Bostancı Mah. Emanet Sok. No: 5 D:6 34744 Kadikoy – Istanbul / Turkey
e-mail: [c.n.emeklioglu@gmail.com](mailto:c.n.emeklioglu@gmail.com)

Cagdas Nurettin Emeklioglu, MD

Orcid: 0000-0003-1859-8680

[c.n.emeklioglu@gmail.com](mailto:c.n.emeklioglu@gmail.com)

Mirac Ozalp, Assoc. Prof

[ozalpmirac@gmail.com](mailto:ozalpmirac@gmail.com)

Orcid: 0000-0002-2255-1642

Elif Akkoc Demirel, MD

[akkocelif@hotmail.com](mailto:akkocelif@hotmail.com)

Orcid: 0000-0003-1910-9113

Simten Genc, Assoc. Prof

[simtengenc@yahoo.com.tr](mailto:simtengenc@yahoo.com.tr)

Orcid: 0000-0003-4446-4467

Veli Mihmanli, Prof

[velimihmanli@yahoo.com](mailto:velimihmanli@yahoo.com)

Orcid: 0000-0001-8701-8462
