# Supplementary material for: Evaluation of the SII and HALP scores in predicting perinatal outcomes in pregnancies with preterm premature rupture of membranes
Source: BMC Pregnancy Childbirth. 2026 Apr 20;26:595. doi: 10.1186/s12884-026-09127-9 (PMC13227662; doi:10.1186/s12884-026-09127-9)
Supplement: Supplementary file 2 — Supplementary Material 2. [file 12884_2026_9127_MOESM2_ESM.docx]

**Dear Editors,**

We are pleased to submit our manuscript entitled **"Evaluation of SII and HALP Scores in Predicting Perinatal Outcomes in Pregnancies with Preterm Premature Rupture of Membranes (PPROM)"** for your consideration for publication in BMC Pregnancy and Childbirth*.*

Preterm premature rupture of membranes (PPROM) remains a major contributor to preterm birth and neonatal morbidity. Identifying practical, accessible, and cost-effective biomarkers to predict neonatal outcomes and delivery timing is critical for improving clinical management. In this retrospective cohort study of 317 PPROM cases, we evaluated the prognostic value of two hematological inflammation-based indices: the **Systemic Immune-Inflammation Index (SII)** and the **Hemoglobin-Albumin-Lymphocyte-Platelet (HALP)** score.

Our findings show that elevated SII values are significantly associated with adverse neonatal outcomes and can accurately predict delivery within seven days following membrane rupture. In contrast, the HALP score demonstrated limited predictive value in this context. These results suggest that SII may serve as a simple and useful tool for risk stratification in pregnancies complicated by PPROM.

To the best of our knowledge, this is the first study to assess the HALP score in PPROM and among the largest to evaluate the SII in relation to perinatal outcomes. We believe our results will be of particular interest to clinicians and researchers focused on improving outcomes in high-risk pregnancies.

This manuscript has not been published or submitted elsewhere. All authors have read and approved the final version and consent to its submission *to*BMC Pregnancy and Childbirth. Ethical approval was obtained from the Ethics Committee of Prof. Dr. Cemil Tascioglu City Hospital (Decision date-number: 06.05.2024, 102), and the study was conducted in accordance with the Declaration of Helsinki. The authors declare no competing interests.

Thank you for your time and consideration.

Sincerely,

Cagdas Nurettin Emeklioglu, MD
Division of Perinatology, Department of Obstetrics and Gynecology, Marmara University, Istanbul, Turkey

[c.n.emeklioglu@gmail.com](mailto:c.n.emeklioglu@gmail.com)
